# Challenges of Cochlear Implantation in Intralabyrinthine Schwannoma Patients: Surgical Procedures and Auditory Outcome

**DOI:** 10.3390/jcm10173899

**Published:** 2021-08-30

**Authors:** Sophia Marie Häussler, Agnieszka J. Szczepek, Stefan Gräbel, Heidi Olze

**Affiliations:** 1 Department of Otorhinolaryngology, Head and Neck Surgery, Campus Virchow-Klinikum, Charité-Universitätsmedizin Berlin, 13353 Berlin, Germany; stefan.graebel@charite.de; 2 Department of Otorhinolaryngology, Head and Neck Surgery, Campus Charité Mitte, Charité–Universitätsmedizin Berlin, 10117 Berlin, Germany; agnes.szczepek@charite.de

**Keywords:** schwannoma, intralabyrinthine schwannoma, microsurgical resection, cochlear implantation

## Abstract

Intralabyrinthine schwannoma (ILS) is a rare benign tumor of the inner ear potentially causing unilateral sensorineural hearing loss and vertigo. This study evaluated the outcome of one surgical session comprising microsurgical ILS resection and cochlear implantation in terms of surgical feasibility, complications, and auditory outcome. Ten clinically and histologically confirmed ILS patients included in this study (three women and seven men; mean age 56.4 ± 8.6) underwent surgery between July 2015 and February 2020. Eight patients had intracochlear tumor location; the remaining two had vestibulocochlear and intravestibular ILS. One of the three following methods was used for tumor removal: an extended cochleostomy, subtotal cochleoectomy, or a translabyrinthine approach. Although negligible improvement was observed in two of the patients, two patients were lost to follow-up, and one opted out from using CI, the speech perception of the five remaining ILS patients improved as per the Freiburg Monosyllable Test (FMT) from 0% before surgery to 45– 50% after the implantation. Our study supports the presented surgical approach’s feasibility and safety, enabling tumor removal and hearing restoration shortly after surgery.

## 1. Introduction

Schwann cells are the main glia of the peripheral nervous system, structurally and physiologically supporting the neurons [1]. Upon neoplastic transformation, the Schwann cells give rise to neurofibromas and schwannomas. Schwannomas are grade I benign neoplasms representing the most common, non-malignant nerve sheath tumor [2]. A typical example of a schwannoma is a vestibular schwannoma (VS, a.k.a. *acoustic neuroma*), which develops on the 8th cranial nerve. The symptoms associated with VS include hearing loss, vertigo, and tinnitus. The hearing loss induced by sporadic VS is unilateral and can be gradually progressive or sudden. Regarding the latter, VS accounts for approximately 3.0–3.4% [3,4] of sudden sensorineural hearing loss (SSHL) cases. The overall incidence of VS in the general population ranges between 1.7 and 4.2 cases per 100,000 [5,6,7,8].

A rare form of schwannoma, described for the first time more than a hundred years ago [9], is the intralabyrinthine schwannoma (ILS) developing inside the inner ear. Salzman et al. [10] classified the ILS based on the tumor location as intracochlear, intravestibular (with or without the involvement of semicircular canals), vestibulocochlear, transmodiolar, transmacular, or transotic. The reported incidence of ILS varies between 0.81 and 1.1 cases per 100,000. However, in recent years, the diagnosis rate of ILS has increased due to improved imaging methods and increasing public awareness regarding hearing impairment [5,11]. Patients with ILS typically present with unilateral sensorineural hearing loss (SHL), which can be progressive and lead to unilateral deafness. In the most extensive study of 110 ILS cases, Dubernard et al. [12] found that sensorineural and progressive hearing loss is the predominant symptom of ILS (94.5%). However, hearing loss as the first symptom of ILS can occasionally fluctuate or occur suddenly, the latter being typical for intracochlear and intravestibular ILS. The presumed mechanisms of hearing loss induced by ILS are compression of the cochlear nerve, damage to the organ of Corti, and damage to the cochlear vasculature, which might reduce the numbers of hair cells and spiral ganglion neurons [13]. In addition to SHL, several patients also report tinnitus and episodes of vertigo [12,14,15].

Therapeutic options for the ILS include passive monitoring, called “wait-and-scan,” and active intervention to remove the tumor. The “wait-and-scan” monitoring uses imaging with a gadolinium contrast agent-enhanced cranial MRI (cMRI) to control tumor growth. Usually, imaging is conducted every six months during the first two years, after which once a year is recommended for the patients with the stable, not progressing disease. The active interventions use a transmastoid approach, stereotactic radiosurgery, or fractioned radiotherapy. The choice of approach depends on the ILS location, the patient’s general state of health, and the individual burden of disease.

Because of the tumor-induced hearing impairment, ILS patients undergo auditory rehabilitation with either a hearing aid, a contralateral-routing of signals (CROS) device, or a cochlear implant (CI). The therapy choice is based on the residual hearing level and the therapeutic approach chosen. For ILS patients with unilateral severe to profound hearing loss, the standard care includes auditory rehabilitation using cochlear implantation. 

Cochlear implantation is a well-established surgical method that improves speech perception, binaural hearing, health-related quality of life, and reduces tinnitus percept [16,17,18]. The implantation procedure is increasingly performed in patients with single-sided deafness (SSD) and asymmetric hearing loss (AHL) [19,20]. Thus, CI can also be considered for ILS patients with SSD or AHL to allow binaural hearing rehabilitation, improve directional hearing, and hearing in noise. In agreement with that, Kronenberg et al. [21], who incidentally found an intracochlear schwannoma during scheduled cochlear implantation, instead of implanting CI with the posterior tympanotomy approach, performed resection of the ILS. Three years later, the patient who had no tumor regression underwent cochlear implantation.

The first resection of translabyrinthine ILS combined with simultaneous cochlear implantation was reported in 2003 [22]. Schutt et al. [23] described an intralabyrinthine ILS resection using an extended round window approach and simultaneous implantation during one surgical session. In Germany, the ILS tumor removal by subtotal cochleoectomy or an extended cochleostomy approach and concomitant cochlear implantation was introduced in 2017 by Plontke et al. [24,25] and Aschendorff et al. [26].

The present study evaluated the feasibility of performing ILS tumor resection and cochlear implantation during the same surgical session. The auditory outcomes of rehabilitation with CI were analyzed depending on different ILS tumor locations and various surgical approaches.

## 2. Materials and Methods

The local Ethics Committee approved the study (appl. no.: EA2/030/13). Written informed consent was obtained from all patients.

Three female and seven male patients with ILS (mean age 56.4 ± 8.6) were consecutively included in this study. Secondary care specialists referred all these patients to our tertiary Cochlear Implantation Unit for hearing rehabilitation with cochlear. ILS was detected with cMRI during the pre-implantation diagnostics.

Diagnostics included collecting a focused medical history and an ear, nose, and throat examination. The auditory performance of all patients was assessed using pure tone audiometry (PTA) and the Freiburg Monosyllabic Test (FMT) performed in a sound booth at 65 dB sound pressure level (SPL). Before surgery, the PTA and FMT were conducted with patients wearing a hearing aid, whereas with a speech processor after the surgery. In addition, auditory brainstem responses (ABR), vestibular examination, and bedside head impulse test (bHIT) were performed before surgery. In patients with a history of vertigo, caloric testing and videonystagmography were added to the test battery. Imaging included cranial magnetic resonance imaging (cMRI) and computer tomography (CT) scan of the temporal bone. cMRI was acquired with 1 mm slice thickness using intravenous gadolinium contrast. The cMRI protocol included T1 and T2 weighted sequences and included additionally T1 vibe fat-saturated post gadolinium sequences, T2w SPACE sequences, and/or 3D CISS sequences.

**Figure 1:**
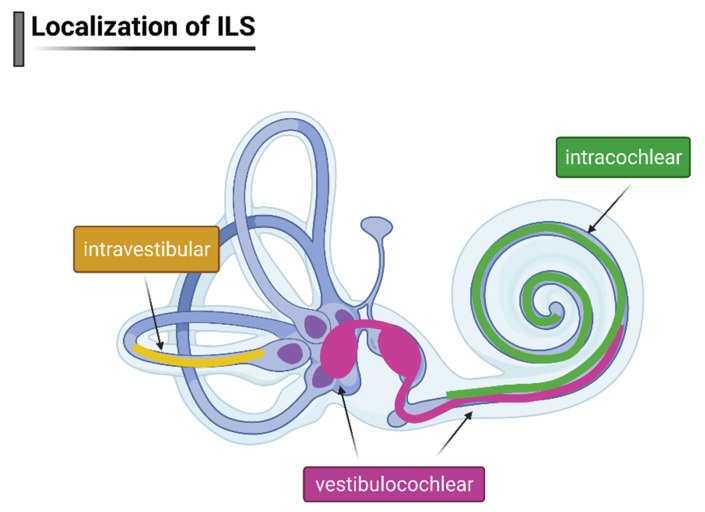
Schematic representation of tumor location (intracochlear, intravestibular, and vestibulocochlear) among ILS patients included in the study. Created with BioRender.com (accessed on 14 July 2021).

The cMRI-imaging identified tumor location as intralabyrinthine in all ten cases. According to Salzmann classification (14), three types of schwannomas were present in the sample: intracochlear, intravestibular, and vestibulocochlear (Figure 1).

Inclusion criteria for the study were:

- age (older than 18)
- absence of neurofibromatosis (using clinical and molecular-pathological methods)
- cMRI-assisted diagnosis of intralabyrinthine schwannoma
- fulfillment of CI indication criteria according to the accepted German guidelines for cochlear implantation

The aim of cochlear implantation was binaural hearing rehabilitation. The patients in this study were diagnosed with two types of hearing impairments: either single-sided deafness (SSD) or asymmetric hearing loss (AHL). SSD was defined as a profound hearing impairment in the affected ear and a functional hearing in the contralateral ear. The loss of hearing threshold in the contralateral ear did not exceed 30 dB in the pure tone average (500 Hz, 1000 Hz, 2000 Hz, and 4000 Hz). AHL was defined as a profound hearing impairment in the poorer ear and a hearing loss in the contralateral ear of more than 30 dB in the four-frequency pure tone average, with a speech perception better than 50% when tested with a hearing aid.

The interdisciplinary board (otologists, neurosurgeons, and radiotherapists) discussed the treatment options with each patient individually. Each time, the option “wait-and-scan” was brought forward. All patients included in the study decided on tumor resection combined with cochlear implantation during the same surgical session. 

Multichannel cochlear implants were supplied by MED-El (Synchrony Flex 28, MED-El, Innsbruck, Austria) or by Cochlear (Nucleus CI 512, CI 532, CI 612, Cochlear, Sydney, Australia). The surgical procedures were performed between July 2015 and February 2020.

## 3. Results

### 3.1. Characterization of ILS Onset and Audiovestibular Symptoms

The first symptom of ILS was sudden unilateral hearing loss in nine of the patients, whereas one patient reported tinnitus as a first symptom. Before surgery, five patients had tinnitus and five intermittent vertigo (including patient #6 with an intravestibular schwannoma).

Six patients were diagnosed with asymmetric hearing loss (AHL), and four with single-sided deafness (SSD). Seven ILS tumors were located in the left ear and three in the right ear.

All patients underwent vestibular examination. Five patients (# 1, 3, 4, 5, and 7) had a history of vertigo, whereas the other five were vertigo-free. Extended vestibular function testing was conducted with Frenzel goggles, bedside Head Impulse Test (bHIT), videonystagmography (VNG), and caloric testing. Two patients had a total vestibular loss, two had a vestibular weakness, and one had a normal vestibular function.

### 3.2. ILS Location

The most common tumor location (*n* = 8) was intracochlear: two patients had an ILS in the basal cochlear turn, three in the middle cochlear turn, in two patients the ILS reached from the basal to the middle cochlear turn and in one patient from the basal to the apical turn (Table 1, Figure 1). One ILS tumor was located in the vestibulocochlear part (patient #1), and another one was located in the intravestibular part of the inner ear (patient #6).

### 3.3. Surgical Approach

The surgical approach depended on the location of the ILS, and the following points were crucial for the planning of the approach: the affected turn of the cochlea, anterior or posterior side of the modiolus, location in the vestibule or semicircular canal, and the size of the tumor. In some cases, it was considered removing the incus and stapes crura might be necessary for a broader approach to the cochlea and better exposure of the ILS.

In the patients who had basal turn-ILS, an extended cochleostomy approach with posterior tympanostomy was used for tumor removal and implantation. Extended cochleostomy was defined as an extended round window approach combined with re-sectioning of the promontory/cochlear wall parts.

For the patients with schwannoma in the middle and basal to apical cochlear turn, a subtotal cochleoectomy approach was used (Table 2), defined as removal of the promontory and parts of the cochlear and parts of the modiolus, necessary to achieve the best access to the tumor mass. Resected parts of the cochlea were reconstructed with fascial tissue, temporal muscle, cartilage, and fibrin glue to prevent postoperative perilymph fistula and dislocation of the CI electrode.

### 3.4. Cochlear Implantation

The patients were implanted with various CI models (Table 2). Four patients received CI 512, three CI 612, and one CI 532 (Cochlear, Sydney, Australia). Two patients opted for MedEl Synchrony Flex 28 (MED-El Innsbruck, Austria). The concomitant resection of the ILS and cochlear implantation took on average 180.4 ± 32.7 min. The same experienced ENT surgeon performed all surgeries.

### 3.5. Tumor Histopathology

Parts of the resected tumor tissues were sent to the Department of Pathology. Histopathological findings revealed compactly arranged spindle cells with zones of increased intercellular matrix. There were no atypical cells, no mitotic figures were found, and the Ki67-proliferation index was less than 1%, confirming the benign character of the tumor. In addition, the spindle cells strongly expressed S100, characteristic of schwannomas and neurofibromas, and used as an exclusion criterion for malignant peripheral nerve sheath tumors [27].

### 3.6. Auditory Rehabilitation

The implantation procedure was surgically successful in all cases. Six patients reported daily CI use and benefits regarding improved speech perception when using the CI speech processor. Speech perception was measured using the FMT test before and after surgery (one, six, 12, and 24 months; Table 2).

Patient #5 did not speak German or English; thus, only the Freiburg two-digit numbers test at 65 dB could be performed. One year after surgery, that patient understood 50% of the two-digit numbers (pre-operatively: 0%).

Six months after the surgery, the FMT indicated an improvement in the auditory abilities of ILS CI-implanted patients: 28% ± 16% (minimum = 5%, maximum = 50%; *n* = 7). Patients with basal turn intracochlear ILS had better auditory outcomes than the others. ILS tumor excision was performed in all these cases via the extended cochleostomy approach (Table 2). The mean score of the FMT in that subgroup improved significantly from 0% preoperatively to 46.6 ± 2.8% postoperatively. The speech perception of patient #6, who had a tumor in the horizontal semicircular canal, also improved (0% before surgery and 50% 24 months after surgery).

Two patients were lost to follow-up.

### 3.7. Complications and Their Treatment

Complications were seen in patient #6, who underwent a translabyrinthine approach to remove ILS from the horizontal semicircular canal. That patient developed postoperative temporary peripheral facial paralysis (House–Brackmann score IV). Patients #9 and #10 had vertigo with spontaneous nystagmus to the contralateral side.

Facial paralysis was treated with a prednisone tapering scheme and physiotherapy. Three months after surgery, the function of the facial nerve recovered. Patients with vertigo were also treated with a prednisone tapering scheme and physiotherapy that included balance training. Vertigo subsided two to three weeks after surgery.

### 3.8. Detailed Description of Five Cases (Cases# 1, 3, 4, 6, and 9)

- Case #1

**Figure 2:**
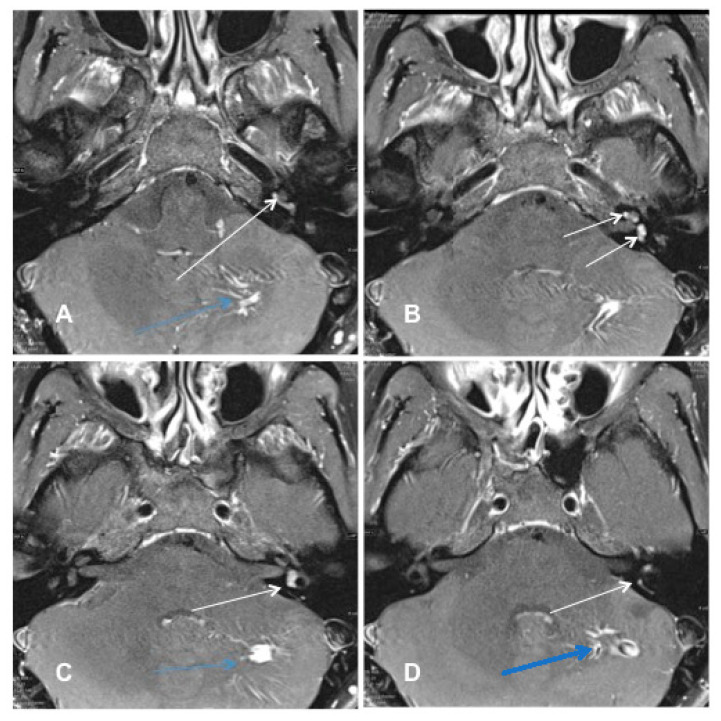
cMRI imaging (Case #1). Shown are T1 weighted images with i.v. gadolinium, fat-saturated, 2 mm. The tumor had a vestibulocochlear extension from the basal turn of the cochlea to the vestibulum and ampulla of the superior and horizontal semicircular duct and their common crus. Blue arrow: venous cerebellar malformation. (**A**) Contrast-enhanced visualization of ILS in the basal cochlear turn (white arrow). (**B**) ILS in the basal cochlear turn and the ampulla of superior/horizontal semicircular duct (white arrow). (**C**) ILS in the ampulla of superior/horizontal semicircular duct (white arrow). (**D**) ILS in the common crus of the superior and horizontal semicircular duct (white arrow).

Patient #1 had a vestibulocochlear type of ILS. The tumor extended from the basal cochlear turn towards the vestibulum, ampulla of the superior and horizontal semicircular duct, and the common crus (Figure 2A–D). Tumor resection was performed with an extended round window and a translabyrinthine approach.

- Case #3

**Figure 3:**
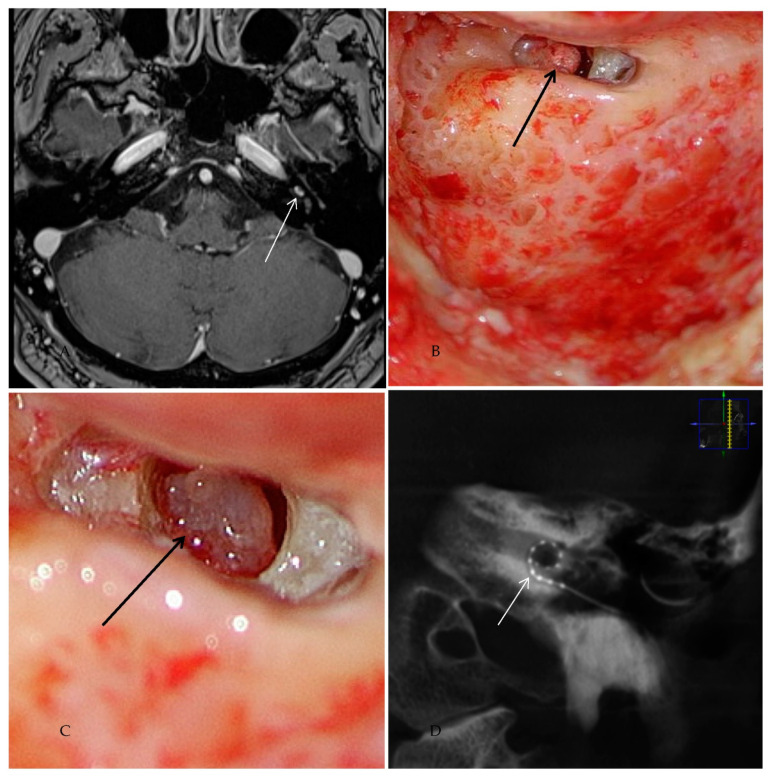
Case #3: ILS of the middle cochlear turn (**A**) MR image T1 VIBE with i.v. gadolinium: the white arrow points to the intracochlear schwannoma in the middle turn. (**B**) Intraoperative image of the subtotal cochleoectomy exposing the schwannoma (black arrow) in the middle turn. (**C**) Magnified image of the intracochlear schwannoma in the middle turn (black arrow). (**D**) Postoperative ConeBeam CT of the CI electrode (white arrow) in the cochlea.

Patient #3 had a middle cochlear turn ILS (Figure 3) requiring subtotal cochleoectomy, meaning that parts of the modiolus had to be removed. One year after ILS removal and cochlear implantation, this patient scored 50% in FMT.

- Case #4

**Figure 4:**
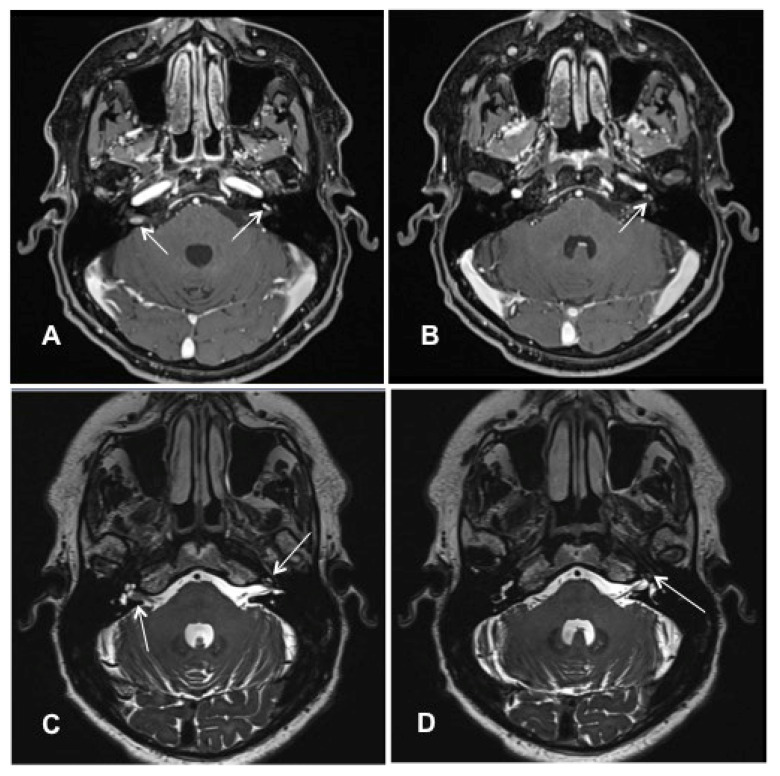
Case #4: MR images visualizing bilateral ILS (right side: intrameatal location, left side: intracochlear location). (**A**,**B**): T1 weighted VIBE 3D with fat saturation prepulse after the intravenous administration of gadolinium. (**A**) White arrows: intrameatal VS on the right side and ILS on the left side. (**B**) The white arrow points to the intracochlear schwannoma on the left side (middle turn); (**C**,**D**): T2 weighted SPACE sequences. (**C**) White arrows point to the intrameatal VS on the right and the ILS on the left. (**D**) The white arrow points at the ILS on the left side (middle turn).

Patient #4 had an ILS of the middle cochlear turn of the left ear and intrameatal vestibular schwannoma of the right ear. Molecular genetic testing and clinical examination excluded neurofibromatosis type 2. cMRI visualized the ILS tumor mass on the left side and an intrameatal VS on the right side (Figure 4A–D). The patient opted for subtotal cochleoectomy with simultaneous cochlear implantation. Removing the incus and the crura of the stapes was necessary for optimal access to the cochlea during the surgery.

- Case #6

**Figure 5:**
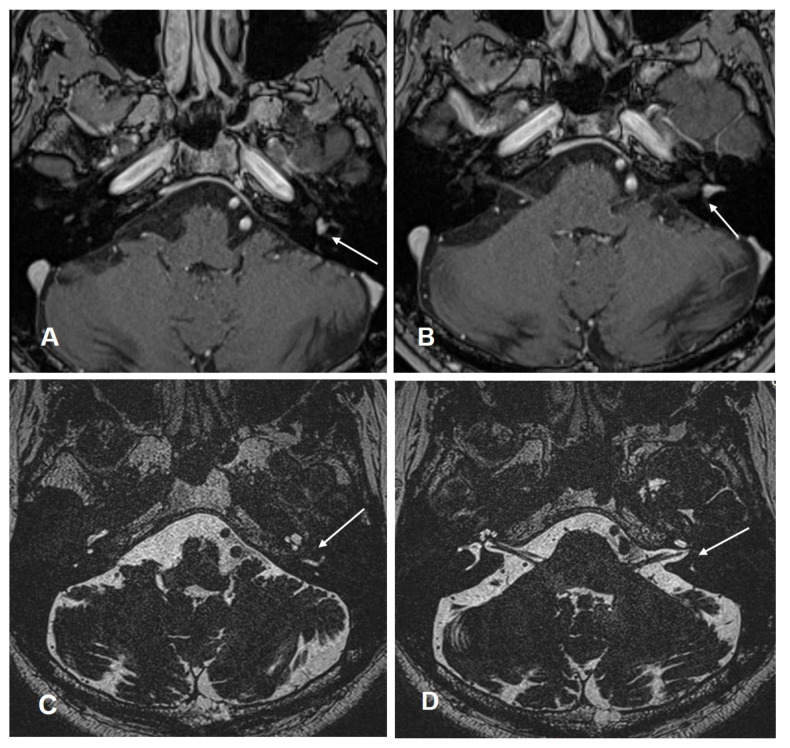
Case #6: cMRI images demonstrating the intravestibular schwannoma. (**A**,**B**): T1 weighted VIBE 3D with fat saturation prepulses after the intravenous administration of gadolinium. White arrow: intravestibular schwannoma in the ampulla and in the anterior part of the horizontal semicircular canal. (**C**,**D**): T2 weighted SPACE sequences. (**C**) Intravestibular schwannoma in the anterior part of the horizontal semicircular canal (white arrow). (**D**) ILS in the ampulla (white arrow).

Patient #6 had intravestibular ILS (in the anterior part of the horizontal semicircular canal, Figure 5A–D). Therefore, it was necessary to use a translabyrinthine approach during tumor removal. For implantation, the round window approach was used. After surgery, the patient developed a transient facial nerve paralysis (House–Brackmann score IV) treated with intravenous prednisone, as per local SOP (dosage during surgery 250 mg; after surgery-tapering scheme down to 5 mg a day) and targeted physiotherapy. Four months after surgery, the facial nerve function was restored.

- Case #9

**Figure 6:**
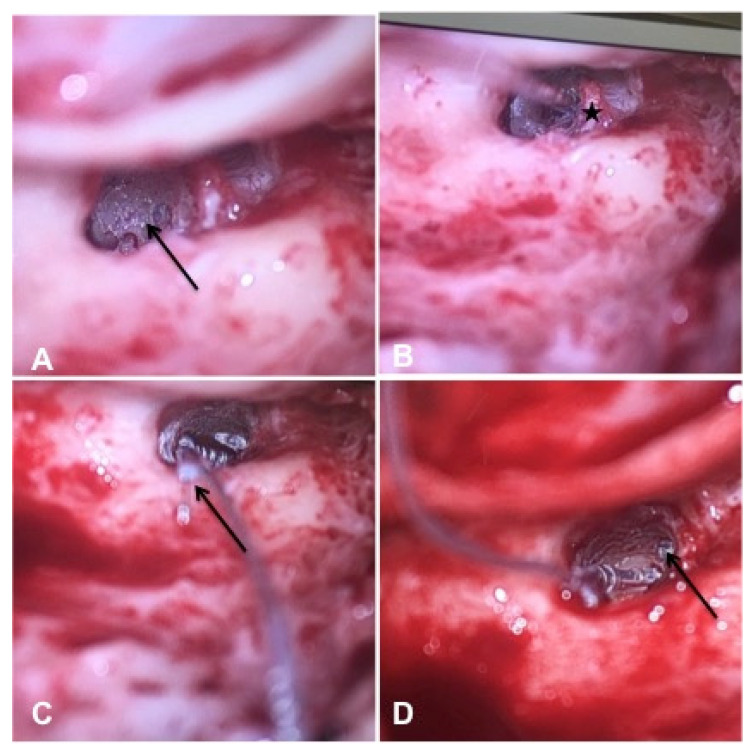
Case # 9: Intraoperative images demonstrating ILS-tumor resection surgery from the basal turn of the cochlea and cochlear implantation. (**A**) ILS in the basal turn after removing the promontory wall and bony structures around the round window (black arrow). (**B**) Resection of the ILS (asterisk marks the cochlear modiolus). (**C**) Insertion of the cochlear implant CI 612 (black arrow marks the stylet of the electrode). (**D**) Image after complete insertion of the electrode. The tip of the electrode can be seen in the middle turn (black arrow).

Patient #9 had basal turn ILS. The tumor mass extended from the lateral wall to the beginning of the middle cochlear turn. Therefore, an extended cochleostomy approach was used for the tumor excision. After ILS removal, insertion of the cochlear implant electrode was performed successfully (Figure 6).

## 4. Discussion

This report describes ten cases with a sporadic ILS, their diagnosis, therapy, and the outcome of auditory rehabilitation with CI implanted during tumor resection. The microsurgical removal of ILS and concomitant cochlear implantation procedures performed in this study were surgically successful in all cases. Resected parts of the cochlea were reconstructed with fascial tissue, temporal muscle, cartilage, and fibrin glue to prevent the CI electrode from dislocation.

In five ILS patients, speech perception measured with FMT improved from 0% to 45–50%; the other patients had either mild improvement (2 patients), were non-users (1 patient), or were lost to follow-up (2 patients). Interestingly, patient #3, who underwent subtotal cochleoctomy, scored 50% in FMT 12 months after surgery, indicating that not the whole modiolus is needed to improve speech perception scores with CI. Our observation corroborates an earlier description of this phenomenon reported by Plontke et al. [28].

Nine of ten patients included in our study had unilateral hearing loss as the first symptom of ILS. That agrees with an extensive review (*n* = 262 of ILS cases) [13] and a multicenter review (*n* = 110 of ILS cases) [12] reporting severe hearing loss on the affected side as the main ILS symptom. A recently published study [29] describes 20 ILS cases, and in each of them, hearing loss was reported on the affected ear. In contrast, Lee et al. [30] suggested that the most common symptom of ILS is dizziness/vertigo combined with auditory symptoms, followed by isolated hearing loss. The discrepancies between Lee’s observations of ILS first symptoms and all other groups could likely reflect the patient recruitment procedure. In an interesting case report, Lee et al. [31] described symptoms and treatment in a case with ILS of the superior and lateral semicircular canal. That patient had progressive fluctuating hearing loss and episodes of vertigo as the first symptoms.

In our study, eight of ten ILS tumors had intracochlear and two intravestibular/vestibulocochlear locations. These locations corroborate the report of Van Abel et al. [13], suggesting that the intracochlear and intravestibular locations predominate in ILS. Similarly, in their multicenter study (*n* = 110), Dubernard et al. [12] reported that the most common ILS locations were intracochlear (50%), followed by vestibular (19.1%), and diffuse (30.9%). Neff et al. [14] suggested that the basal turn of the cochlea is the most frequent location of ILS, supported by our observation of basal or basal-to-middle turn in five of eight intracochlear cases and a basal location in one case with a mixed location. The remaining three intracochlear tumors were located in the middle turn of the cochlea.

The current treatment options for ILS are microsurgery, radiation therapy (in particular stereotactic radiosurgery), and wait-and-scan. Because of ILS-induced hearing impairment, most patients opt not only for tumor treatment but also for hearing rehabilitation. In the present study, we treated ten ILS patients with ILS-induced hearing loss using microsurgical tumor resection combined with cochlear implantation during the same surgical session. Gosselin et al. and Neff et al. [14,32] also recommend microsurgery to remove ILS tumors for patients with recurrent vertigo and/or deafness. In the present study, microsurgical resection was a treatment of choice. The reason for choosing microsurgery was transmastoidal access, practicable during labyrinthectomy, cochleostomy, or an extended round window approach.

The ILS tumor resection is conducted first during sequential surgery, and cochlear implantation is performed during the second surgery. However, a sequential approach can lead to fibrosis in the inner ear tissues after the first surgery, making the implantation very difficult, if not impossible. Kronenberg et al. [21] reported sequential cochlear implantation three years after ILS removal by cochleostomy approach. Using the posterior tympanotomy, they found connective tissue in the facial recess, which was assumed to be postoperative tissue fibrosis. In addition, the authors found that only ten of twelve apical electrodes could be activated.

To address the problem of fibrosis, Beutner et al. [33] and Aschendorff et al. [26] removed the tumor mass and inserted a dummy electrode into the cochlea to prevent cochlear obliteration and tissue fibrosis. After cMRI imaging and a promontory test, they performed cochlear implantation using a translabyrinthine approach [33] or labyrinthectomy [34]. The advantage of using a dummy electrode is the unrestricted imaging with cMRI to monitor possible recurrence or residual tumor before implantation. Sequential surgery with a dummy electrode presents a good clinical option, in particular for multilocular, vestibulocochlear, transmodiolar, transmacular, or transotic schwannomas. In these types of ILS, total resection is complex, and there is a risk that not the entire tumor mass would be evacuated. However, no study to date had addressed the degree of neuronal degeneration or cochlear obliteration in ILS patients who underwent sequential surgery. Lastly, in contrast to the simultaneous surgery described in the present study, sequential surgery uses two surgical interventions under general anesthesia, and the patient has to be admitted twice to the hospital. Both factors are of importance regarding patients’ health and health economics.

The possible changes in the cochlea occurring during wait-and-scan management of ILS remain unknown. Van Abel et al. [13] favor wait-and-scan despite the danger of continuous tumor expansion from the primary site to other labyrinth parts that might complicate later tumor resection. Additionally, the wait-and-scan method with sequential surgery challenges the well-known correlation between the duration of deafness and the degree of speech perception after cochlear implantation [35]. Carlson et al. [36] reported the option of intentionally leaving intracochlear schwannoma in situ to prevent the trauma to the modiolus and osseous spiral lamina and performed implantation using a stiff CI electrode with stylet for insertion.

Bagattini, Quesnel, and Röösli demonstrated the degree of neurodegeneration caused by untreated ILS [37]. The authors performed histopathological analyses of temporal bones obtained from deceased patients with known medical history. The cause of death was unrelated to ILS. Of 39 sporadic schwannomas identified in that study, six cases were ILSs. The patients were advanced in age at the time of death (range 68–94). In contrast, the hearing and/or vertigo symptoms were reported relatively early in life (22 months to 70 years). The degeneration of spiral ganglion neurons was histologically proven in four of the six cases. It remains, however, unclear how long the tumor had been present in the labyrinths, as the medical history of deafness or vertigo must not necessarily mean the onset of ILS, and the imaging diagnostic was not available. Taken together, one should assume that at least in some of the ILS patients, the tumor mass could have caused neuronal dystrophy. In addition, resection of parts of the inner ear and modiolus likely adds to the damage of nerve fibers in the inner ear.

Nevertheless, our present work and the work of others imply that hearing rehabilitation of ILS patients using CI is a plausible method [28]. In another study, Rahne et al. described recording CI-evoked brainstem responses after removing the intralabyrinthine portion of a schwannoma [38]. Such a technique provides an option for intraoperative functionality assessment of the hearing nerve. Another exciting technique enabling intraoperative monitoring is electrocochleography. It provides additional information about cochlear and neural dysfunctions [39] and could help preserve residual hearing [40,41].

An alternative two-step surgical approach offers the possibility of testing for hearing nerve function. In the first step, tumor removal is performed, and hearing nerve function is tested after surgery with cochleographic and promontory tests. In the second step, cochlear implantation is performed.

Plontke et al. [24,42] described the partial or subtotal cochleoectomy, cochlear implantation, and cochlea reconstruction with temporal muscle fascia, cartilage, perichondrium, and bone pate. We, too, used this technique in this study during subtotal cochleoectomy for the cochlear reconstruction, which is essential to prevent postoperative perilymph fistula [28], dizziness, and dislocation of CI electrode.

In the present study, the audiological outcome measured six months after surgery with FMT at 65 dB SPL varied from 0% to 50 %. The mean value of FMT at 65 dB for ILS patients (*n* = 7) was 28 ± 16%; the mean FMT at 65 dB for the intracochlear ILS (*n* = 5) was 32 ± 14%. In addition to various tumor locations and different surgical approaches to remove the tumor, our cohort consisted of patients with SSD and AHL, making the sample heterogeneous and likely contributing to the wide range of auditory outcomes observed in the study. Kronenberg et al. [21] reported a patient treated with sequential ILS and CI surgery who postoperatively had a score in the syllable testing (Hebrew) of 100%. During the open-set presentation of everyday sentences in Hebrew, that patient scored 30%. Aschendorff et al. [26] described four cases, which ranged between 10% and 95% in FMT (65 dB) six months postoperatively. In their recent comparative analysis, Plontke et al. [28] reported that one year after cochlear implantation, patients with ILS and subtotal cochleoectomy or extended cochleostomy scored better (75% FMT at 65 dB) than patients treated with standard procedure (FMT 58% at 65 dB). One exception was a patient with near-total cochleoectomy, who had a word recognition score of 30% (FMT at 65 dB) six months after surgery. The authors conclude that preserving the basal and the second-turn modiolus is beneficial for the best audiological outcome.

Regarding the adverse effects of the surgery, Plontke et al. [28,42] reported postoperative dizziness in most ILS patients after subtotal cochleoectomy. Still, they also described one case with subtotal cochleoectomy and preserved vestibular function. In the present study, only two of the ten patients had postoperative vertigo and vestibular function loss detected by bedside head impulse test and contralateral spontaneous nystagmus. The vestibular symptoms were successfully treated and subsided a few weeks after surgery.

The major pitfalls of our study include the small sample size, measurement of the auditory outcome in all of the included patients only six months after implantation, heterogeneity of cases regarding tumor location, duration of hearing impairment, and various types of hearing impairments. A very low incidence of ILS accounts for the small sample size, various tumor locations, and the different duration of hearing impairment on the affected ear. In addition, in Germany, the indication for cochlear implantation in patients with AHL and SSD (both types of hearing impairments associated with ILS and diagnosed in our cohort) has been in place only since 2012. Another drawback of our study is that two of the ten ILS patients were lost to follow-up shortly after surgery, and one patient opted out from using the CI. That can only be addressed by extending the sample size or designing a multicenter study. The last pitfall of this study is a lack of information about the degree of neurodegeneration that ILS and surgical intervention could have caused. The functionality of the hearing nerve must be tested before implantation [43], as it is a prerequisite to meet implantation criteria. However, that method does not permit refined stratification of possible neuronal damage, leaving a gap in diagnostic and prognostic methods for ILS patients, which needs to be addressed in the future.

Our present study demonstrates the outcome of auditory rehabilitation of ILS patients who underwent microsurgical ILS tumor removal and concomitant cochlear implantation during the same surgical session. The obtained results support use of the double surgical procedure. The existing studies and case reports about ILS and their treatment are low in number, reflecting a low incidence of ILS, which increased recently, indicating the actual occurrence rate of ILS and improved disease detection. In addition to presenting encouraging results of hearing rehabilitation of ILS patients, our study creates awareness of this rare disease that in the future might become a more common surgical and auditory challenge in clinical ENT practice.

## 5. Conclusions

Our present work demonstrated that one-session microsurgical removal of ILS and cochlear implantation is surgically feasible and safe. Although our results imply that only every second ILS patient benefits concerning speech comprehension from the surgical procedure described, increasing the sample size through a multicenter approach and developing a better follow-up scheme could likely improve this success rate.

## Figures and Tables

**Table 1:** Patients characteristics. *r* = right, l = left; SSD = single sided deafness, AHL = asymmetric hearing loss, DSD = double-sided deafness.

Patient Nr.	Male/Female (m/f)	Age [Years]	Affected Side (*r*/l)	Type of Hearing Loss	Tumor Location (Salzmann et al.)	Max. Tumor Diameter [mm]	Cochlear Turn	First Symptom
1	f	40	l	SSD	vestibulo-cochlear	5.0	basal	hearing loss
2	m	57	r	AHL	intracochlear	3.5	basal	hearing loss
3	m	57	l	AHL	intracochlear	2.8	middle	hearing loss
4	m	55	l	AHL	intracochlear	5.0	middle	hearing loss
5	f	72	r	AHL	intracochlear	5.5	basal to apical	hearing loss
6	m	54	l	AHL	intravestibular	3.0	-	hearing loss
7	m	56	l	SSD	intracochlear	4.5	basal to middle	hearing loss
8	m	64	r	AHL	intracochlear	3.0	basal	hearing loss
9	f	48	l	SSD	intracochlear	6.5	basal to middle	hearing loss
10	m	61	l	SSD	intracochlear	5.5	middle	tinnitus

**Table 2:** Surgical approaches and speech perception in the operated ear following tumor resection and cochlear implantation. FMT = Freiburg Monosyllable Test.

Patient #.	Date of CI (Month/Year)	CI-Model	Surgical Approach	FMT Pre CI (%)	FMT 1 Month Post C (%)	FMT 6 Months Post CI (%)	FMT 12 Months Post CI (%)	FMT 24 Months Post CI (%)
1	07/2015	CI 512	translabyrinthine/extended cochleostomy	0	5	5	Non-user	-
2	07/2015	CI 512	extended cochleostomy	0	0	20	45	-
3	10/2017	MedElSF 28	subtotal cochleoectomy	0	-	25	50	-
4	10/2017	MedElSF 28	subtotal cochleoectomy	0	5	20	15	0
5	12/2017	CI 512	subtotal cochleoectomy	0	-	-	-	-
6	04/2018	CI 532	translabyrinthine/ round window	0	15	35	10	50
7	06/2019	CI 512	subtotal cochleoectomy	0	-	-	-	-
8	10/2019	CI 612	extended cochleostomy	0	-	50	-	-
9	10/2019	CI 612	extended cochleostomy	0	5	45	-	-
10	02/2020	CI 612	subtotal cochleoectomy	0	10	-	-	-

## Data Availability

Data are available upon request.
